# Primary Neuroendocrine Tumor in Brain

**DOI:** 10.1155/2014/295253

**Published:** 2014-11-20

**Authors:** Ryota Tamura, Yoshiaki Kuroshima, Yoshiki Nakamura

**Affiliations:** Department of Neurosurgery, Tokyo Medical Center, 2-5-1 Higashigaoka, Meguro-ku, Tokyo 152-8902, Japan

## Abstract

The incidence of brain metastases for neuroendocrine tumor (NET) is reportedly 1.5~5%, and the origin is usually pulmonary. A 77-year-old man presented to our hospital with headache and disturbance of specific skilled motor activities. Computed tomography (CT) showed a massive neoplastic lesion originating in the left temporal and parietal lobes that caused a mass edematous effect. Grossly, total resection of the tumor was achieved. Histological examination revealed much nuclear atypia and mitotic figures. Staining for CD56, chromogranin A, and synaptophysin was positive, indicating NET. The MIB-1 index was 37%. Histopathologically, the tumor was diagnosed as NET. After surgery, gastroscopy and colonoscopy were performed, but the origin was not seen. After discharge, CT and FDG-PET (fluoro-2-deoxy-d-glucose positron emission tomography) were performed every 3 months. Two years later we have not determined the origin of the tumor. It is possible that the brain is the primary site of this NET. To our knowledge, this is the first reported case of this phenomenon.

## 1. Introduction

Neuroendocrine tumors (NETs) are neoplasms derived from nerve and endocrine cells. NETs have the ability to produce hormones and have similarities with nerve cells, such as cytoplasmic granules and exocytotic machinery. NETs were called “carcinoid” 100 years ago and were considered benign neoplasms. Currently they are considered to be malignant, and the WHO histopathological classification eliminated the “carcinoid” label in 2000. In the WHO classification of 2010, NETs were defined as neuroendocrine neoplasms and were classified as NET G1, NET G2, NEC (large cell or small cell type), mixed adenoneuroendocrine carcinoma (MANEC), hyperplastic, and preneoplastic lesions, by the Ki-67 index [1–4]. NETs are increasing and are therefore attracting interest and attention. Generally, the majority of metastases occur in the liver, lungs, and bone. Other sites are rarer, and brain metastases are very rare. Here we present a case report of a probable primary brain NET. To our knowledge, this is the first reported case of a primary NET arising in that anatomical location.

## 2. Case Presentation

**Figure 1:**
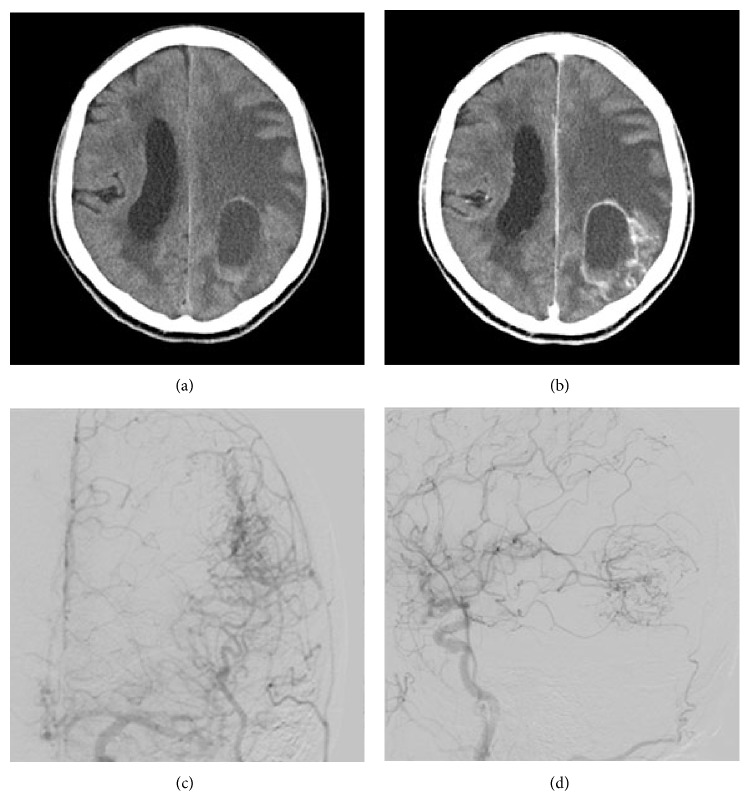
(a) Axial nonenhanced CT scan on admission shows a sharply marginated mass of 6.6 cm in diameter in the left temporal and parietal lobes and compressing the surrounding parenchyma. The left lateral cerebral ventricle is compressed. The lesion had a high-density region in the dorsal aspect indicating hemorrhage. (b) Axial enhanced CT image shows a massive lesion like a cyst with enhancement. (c) Digital subtraction angiography (DSA) of the frontal view shows tumor staining via the middle cerebral artery. (d) DSA of the lateral view shows tumor staining and no arteriovenous shunts.

**Figure 2:**
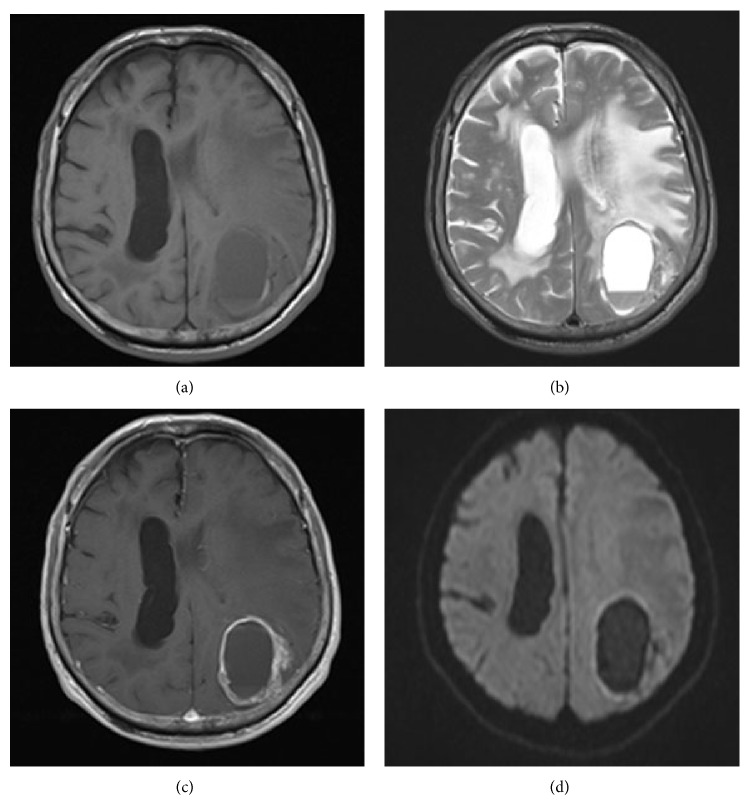
(a) Axial plain T1-weighted MR image shows homogeneous low-intensity area in the left temporal and parietal lobes. (b) Axial plain T2-weighted MR image shows a high-intensity area that indicates hemorrhage, a cyst, and marked peritumoral edema. (c) Diffusion-weighted image (DWI) shows low-intensity area not indicative of abscess. (d) Axial enhanced T1-weighted MR image shows ring enhancement.

**Figure 3:**
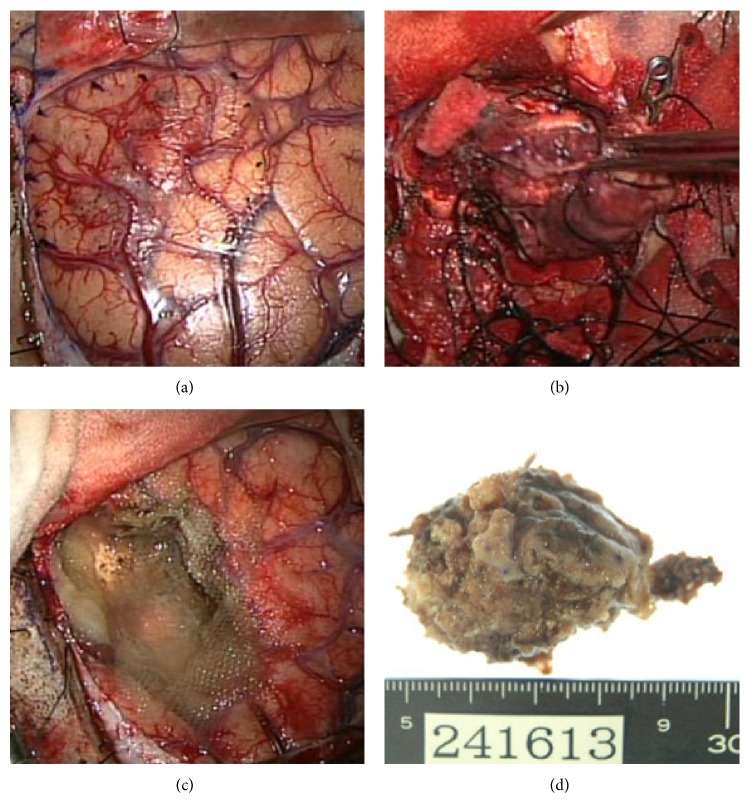
(a) The brain surface was yellow and had abnormal neovascularization. (b) The tumor bled easily and had some cysts and areas of old hemorrhage. (c) The tumor was resected totally in a procedure similar to gyrectomy. 5-ALA was used to assess resection. (d) Exenterated specimen was yellowish and 5 cm in diameter.

**Figure 4:**
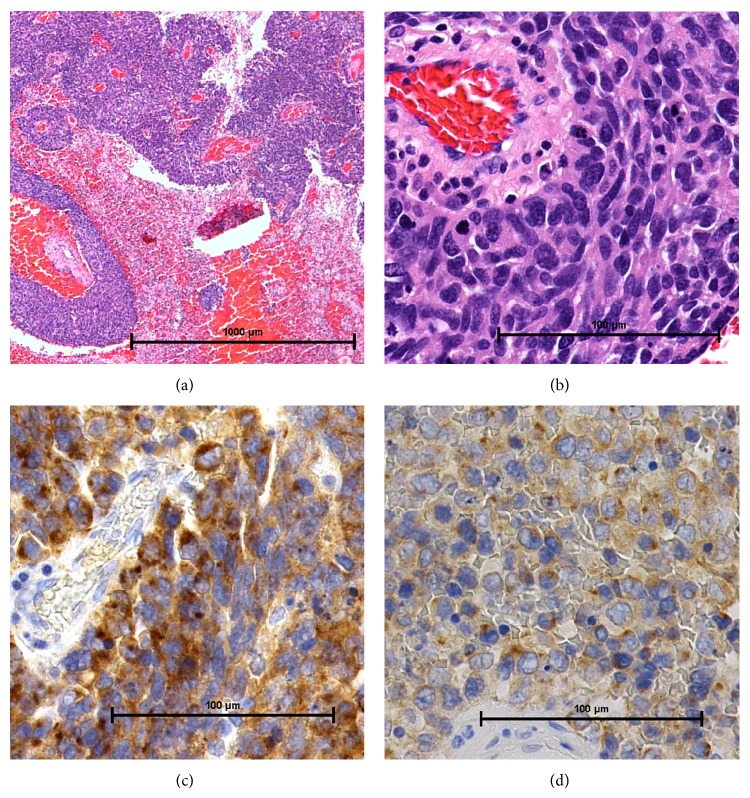
(a) Photomicrograph showing the tumor filled with necrosis. (H&E stain, original magnification ×20. Bar: 1000 *μ*m.) MIB-1 index, 37%. (b) Photomicrograph showed a large number of cells with nuclear atypia around the vessels. (H&E stain, original magnification ×100. Bar: 100 *μ*m.) (c) Chromogranin stain is positive. (Original magnification ×200. Bar: 100 *μ*m.) (d) Synaptophysin stain is positive. (Original magnification ×200. Bar: 100 *μ*m.)

A 77-year-old man presented with headache and disturbance of skilled motor activities. The past medical history included hypertension, diabetes, and chronic idiopathic pericardial effusion. On physical examination, the Glasgow Coma Scale (GCS) score was 14 (E4V4M6), and finger agnosia and left-right disorientation were noted, like in the Gerstmann syndrome. Blood and CSF analyses were normal. Levels of tumor markers, including CEA, CA19-9, NSE, AFP, and IL-2R, were normal. Head computed tomography (CT) showed a massive neoplastic lesion of 6.6 cm in diameter originating in the left temporal and parietal lobes that caused a mass edematous effect. That lesion included a high-density area in the dorsal aspect that indicated hemorrhage (Figure 1(a)). Enhanced CT showed ring enhancement (Figure 1(b)). Head magnetic resonance imaging (MRI) showed a subcortical lesion that contained a cyst and hemorrhage. The central portion was low on T1-weighted imaging (T1WI) and high on T2-weighted imaging (T2WI) (Figures 2(a) and 2(b)). The cyst in the tumor was not enhanced, but other sites were inhomogeneously enhanced (Figure 2(c)). Diffusion-weighted image (DWI) shows low-intensity area (Figure 2(d)). There were no other neoplastic lesions noted on whole-body CT scanning. Angiographic examination showed tumor staining via the left middle cerebral artery, and no AV shunts were seen (Figures 1(c) and 1(d)). Neuronavigation guided tumor removal was performed after oral administration of 5-aminolevulinic acid (5-ALA) on admission Day 11. The tumor had recruited new blood vessels, like red veins, on the brain surface, and the tumor bled easily (Figure 3(a)). We observed old hemorrhage and yellowish fluid in the cystand completely resected the lesion by gyrectomy (Figures 3(b), 3(c), and 3(d)). After that, we resected using 5-ALA fluorescence as a rider. Histopathologically, the tumor was filled with necrosis, and a large number of cells with nuclear atypia were recognized around the vessels. The atypical cells were characterized by their oval shaped nuclei, high N/C ratio, and markedly increased chromatin. There was a portion where the disposition appeared to be flowing and enlarged nuclear bodies were not seen (Figures 4(a) and 4(b)). On immunostaining, glial fibrillary acidic protein (GFAP) was negative, and cytokeratin-pan AE1/AE3 was positive. For the differential diagnosis, glioblastoma was ruled out, and we considered metastasis of carcinoma. Cytokeratin 7 was positive, cytokeratin 20 was negative, and epithelial membrane antigen (EMA) was positive. Lymphocytic markers were negative. As for endocrine markers, chromogranin A, CD56, and synaptophysin were slightly positive (Figures 4(c) and 4(d)). Also, thyroid transcription factor 1 (TTF-1) was positive on most tumor cells, and surfactant apoprotein was negative. However, thyroglobulin and calcitonin indicating thyroid cancer were negative. CD99 was negative, so primitive neuroectodermal tumor (PNET) was interpreted as negative. Both 34*β*E12 and E-cadherin were negative. Considering the pathology and immunostaining results, a high-grade endocrine carcinoma was thought feasible. Grossly, total resection of the tumor was achieved; this was confirmed by postoperative enhanced CT. The mass effect had decreased, and CT showed a small amount of hemorrhage caused by the operation. After surgery, imaging by gastroscopy and colonoscopy was performed, but a tumor origin was not detected. Also, ultrasonography could not point out the thyroid lesions. Postoperative whole brain radiation therapy (30 Gy) was performed from Day 26 to Day 42. The patient had just a little motor aphasia after surgery and was discharged on Day 57. After discharge from the hospital, CT and FDG-PET (fluoro-2-deoxy-d-glucose positron emission tomography) were performed every 3 months, but a tumor origin outside the brain was not identified. On Day 270, an MRI revealed a small local recurrence with ring enhancement in the left parietal lobe near the operative site. Therefore a second surgical procedure was performed with neuronavigation and use of 5-ALA. We resected broadly, as is done for a gyrectomy. But on Day 570, MRI again revealed the small local recurrence, so gamma knife therapy was performed. From then on, until the writing of this paper (Day 720), the patient has remained free from relapse.

## 3. Discussion

Generally, the majority of NET metastases occur in the liver, lungs, and bone, and involvement of other sites is much rarer. NETs are considered to be the origin of brain metastases in 1.5~5% of all patients with brain metastases, and in 45–71% of these patients, the primary tumor was located in the bronchi or lungs. If brain metastases are present, lymph node metastases are found in 75% and liver metastases are found in 50% of these patients [5–8]. Primary unknown NET represents just 13% of these tumors. It is difficult to detect the primary focus, especially for functional NETs, because patients have specific symptoms when the tumor size is small. Somatostatin receptor scintigraphy (SRS) is useful to detect the primary focus, especially for NET G1 and NET G2, whereas PET is more sensitive. The most widely used tracer is F18-deoxyglucose (FDG), but well-differentiated NETs do not uptake the FDG very well; therefore, 68 Ga-DOTA-TOC PET could be better for detecting pulmonary NETs [9]. Somatostatin receptor imaging, by 111In-pentetreotide scintigraphy or PET with 68 Ga-DOTA-TOC PET, frequently identifies lesions that are not visible on other radiographic images. Currently, somatostatin receptor scintigraphy with 111In-pentetreotide is frequently available technique to determine somatostatin receptor expression. In the future, because of its higher sensitivity, 68 Ga-DOTA-TOC PET is expected to replace somatostatin receptor scintigraphy. However, 68 Ga-DOTA-TOC PET is not available in any place. So FDG-PET is more easy to be performed. FDP-PET is usually sufficient for detection of the tumor if the patient has a long-term follow-up even if the tumor is not high-grade NET.

To our knowledge, there is only one previous report of a possible primary brain NET lesion [10]. The patient, a 35-year-old man, had one larger lesion with mixed solid and cystic components located in the left basal ganglia and thalamus and a second cystic lesion located deep in the right parietal lobe. The lesions had similar ring enhancement. But this patient had multiple lesions, so the conclusion was possible metastases. Our case involved a single lesion and had only local recurrence during a prolonged course. FDP-PET could not detect an origin outside the brain, and tumor markers of possible origin like thyroid cancer were negative. TTF-1 that indicates lung or thyroid cancer was certainly positive. However, it is also said that specificity of TTF-1 is not so high. In our case, as I described in case presentation, thyroglobulin and calcitonin indicating thyroid cancer were negative. Lung cancer is usually detected within the long-term follow-up period with CT and PET, but it was not detected in this case. This is a rare clinical course, and we could not deny the possibility that brain was the primary site. So there is the possibility that brain was the primary site of this NET. To our knowledge, this is the first reported case of this phenomenon. There are some other brain tumors that have an epithelial-like pattern, such as ependymoma, pineal tumor, and choroid plexus adenoma. However, there are no epithelial cells in the brain and NET from a central nerve origin is unlikely. Central nerves become neurocanals in the early stages of development, so presence of ectopic epithelial cells is unlikely; however, it seems there may be some exceptions to that general rule. Making primary site of brain as new concept is so important learning point.

## 4. Conclusions

This is the first reported case of a primary NET arising in brain.

It is possible that aberrant ectopic epithelial cells are found in the brain.
